# Whole-genome and Epigenomic Landscapes of Malignant Gastrointestinal Stromal Tumors Harboring *KIT* Exon 11 557–558 Deletion Mutations

**DOI:** 10.1158/2767-9764.CRC-22-0364

**Published:** 2023-04-24

**Authors:** Keiichi Ohshima, Takeshi Nagashima, Keiichi Fujiya, Keiichi Hatakeyama, Yuko Watanabe, Kimiko Morimoto, Fukumi Kamada, Yuji Shimoda, Sumiko Ohnami, Akane Naruoka, Masakuni Serizawa, Shumpei Ohnami, Hirotsugu Kenmotsu, Akio Shiomi, Yasuhiro Tsubosa, Etsuro Bando, Teiichi Sugiura, Takashi Sugino, Masanori Terashima, Katsuhiko Uesaka, Kenichi Urakami, Yasuto Akiyama, Ken Yamaguchi

**Affiliations:** 1 Medical Genetics Division, Shizuoka Cancer Center Research Institute, Shizuoka, Japan.; 2 Cancer Diagnostics Research Division, Shizuoka Cancer Center Research Institute, Shizuoka, Japan.; 3 SRL, Inc., Tokyo, Japan.; 4 Division of Gastric Surgery, Shizuoka Cancer Center Hospital, Shizuoka, Japan.; 5 Cancer Multiomics Division, Shizuoka Cancer Center Research Institute, Shizuoka, Japan.; 6 Drug Discovery and Development Division, Shizuoka Cancer Center Research Institute, Shizuoka, Japan.; 7 Division of Genetic Medicine Promotion, Shizuoka Cancer Center Hospital, Shizuoka, Japan.; 8 Division of Thoracic Oncology, Shizuoka Cancer Center Hospital, Shizuoka, Japan.; 9 Division of Colon and Rectal Surgery, Shizuoka Cancer Center Hospital, Shizuoka, Japan.; 10 Division of Esophageal Surgery, Shizuoka Cancer Center Hospital, Shizuoka, Japan.; 11 Division of Hepato-Biliary-Pancreatic Surgery, Shizuoka Cancer Center Hospital, Shizuoka, Japan.; 12 Division of Pathology, Shizuoka Cancer Center Hospital, Shizuoka, Japan.; 13 Immunotherapy Division, Shizuoka Cancer Center Research Institute, Shizuoka, Japan.; 14 Shizuoka Cancer Center Hospital and Research Institute, Shizuoka, Japan.

## Abstract

Gastrointestinal stromal tumors (GIST) with *KIT* exon 11 deletions involving in codons 557–558 (*KIT* Δ557–558) exhibit higher proliferation rates and shorter disease-free survival times compared with GISTs with other *KIT* exon 11 mutations. We analyzed 30 GIST cases and observed genomic instability and global DNA hypomethylation only in high-risk malignant GISTs with *KIT* Δ557–558. Whole-genome sequencing revealed that the high-risk malignant GISTs with *KIT* Δ557–558 (12 cases) had more structural variations (SV), single-nucleotide variants, and insertions and deletions compared with the low-risk, less malignant GISTs with *KIT* Δ557–558 (six cases) and the high-risk (six cases) or low-risk (6 cases) GISTs with other *KIT* exon 11 mutations. The malignant GISTs with *KIT* Δ557–558 showed higher frequency and significance in copy number (CN) reduction on chromosome arms 9p and 22q, and 50% of them had LOH or CN-dependent expression reduction in *CDKN2A*. In addition, SVs with driver potential were detected in 75% of them, in which *AKT3* and *MGMT* were recurrently identified. Genome-wide DNA methylation and gene expression analyses showed global intergenic DNA hypomethylation, *SNAI2* upregulation, and higher expression signatures, including p53 inactivation and chromosomal instability, as characteristics of malignant GISTs with *KIT* Δ557–558 that distinguished them from other GISTs. These genomic and epigenomic profiling results revealed that *KIT* Δ557–558 mutations are associated with increased genomic instability in malignant GISTs.

**Significance:**

We present genomic and epigenomic insights into the malignant progression of GISTs with *KIT* exon 11 deletions involving in 557–558, demonstrating their unique chromosomal instability and global intergenic DNA hypomethylation.

## Introduction

Gastrointestinal stromal tumors (GIST) are the most common mesenchymal tumors of the gastrointestinal tract, with a worldwide annual incidence of six to 22 cases per million population (1). GISTs originate from the interstitial cells of Cajal (ICC) or ICC-like stem cell precursors and most commonly affect the stomach (50%–60%), followed by the small intestine (30%–35%), rectum (5%), and esophagus (<1%; ref. 2). They often recur locally within the abdomen and/or metastasize to the liver (3). The prognosis for localized GISTs has been assessed by risk stratification schemes to identify tumors that are more likely to recur at distant sites after curative surgery (1). The modified NIH consensus criteria classify GISTs according to size, mitotic count, tumor location, and tumor rupture (4) and are useful for identifying patients who may benefit from adjuvant therapy (5).

Gain-of-function mutations in *KIT* occur in approximately 70% of GISTs (1). Together with *PDGFRA*, which is mutated in 15% of GISTs, *KIT* encodes a receptor tyrosine kinase gene. These mutually exclusive mutations constitutively activate downstream signaling pathways, including RAS/RAF/MAPK and PI3K/AKT/mTOR, leading to cancerous cells (1, 2). Tyrosine kinase inhibitors (TKI), including imatinib, sunitinib, and regorafenib, are highly effective drugs for GIST treatment (1–3). Pathologic *KIT* mutations, including point mutations, deletions, and insertions, have been observed in exons 8, 9, 11, 13, and 17, with exon 11 mutations accounting for 80%–90% of cases (1). These mutations show a type- and location-specific relationship with risk stratification, clinical manifestations, and drug response (1–3).

GISTs carrying *KIT* exon 11 mutations with deletions at codon 557 and/or 558 have a poorer prognosis than those with mutations at other sites (6–9). Deletions at both codons 557 and 558 promote liver metastasis (10) and are thought to be required for malignant transformation (11). The malignant progression of GISTs has also been associated with chromosomal changes, including deletions in chromosome arms 1p, 13q, 14q, 15q, and 22q; gains in chromosomes 4 and 5 (12–14); and genetic alterations, including mutations, copy-number (CN) abnormalities, and aberrant expression of cell cycle–related genes (*p53*, *CDKN2A*, and *RB1*; ref. 15) and PI3K pathway–related genes (*PIK3CA* and *PTEN*; ref. 16). Epigenomic analysis has identified promoter hypermethylation of cell cycle–related genes (17) and *LINE-1* DNA hypomethylation in high-risk and malignant GISTs (18). However, these results are based on comparative analyses between malignant and less malignant cases, and there are no reports of systematic genomic and epigenomic analysis specifically targeting *KIT* exon 11 deletions involving in codons 557–558 (*KIT* Δ557–558).

Whole-genome sequencing (WGS) is a powerful tool capable of revealing the diversity and complexity of global genomic alterations, such as structural variations (SV) and copy-number alterations (CNA) as well as mutations. Thus, we performed WGS along with genome-wide DNA methylation and gene expression analyses in 30 GIST cases to determine the genomic and epigenomic differences between *KIT* Δ557–558 and other *KIT* exon 11 mutations. This included signature analysis of the expression data, and we discuss the relationship between the hypoxia signature and the recently approved Hsp90 inhibitors (19).

## Materials and Methods

### Clinical Samples

Tumor and matched normal tissue samples along with whole-blood samples were obtained from patients receiving surgical treatment at the Shizuoka Cancer Center who were participants in the Project HOPE multi-omics study (20). The study was approved by the Institutional Review Board of the Shizuoka Cancer Center (authorization number: 25–33). All patients agreed to participate in the study and provided written informed consent. All experiments using clinical samples were performed in accordance with the ethical principles of the Declaration of Helsinki. Table 1 presents clinicopathologic data of the 30 patients with GIST in our study cohort. *KIT* mutations were assessed by whole-exome sequencing (WES), panel sequencing, Sanger sequencing, and the Integrative Genomics Viewer (IGV) tool (16). The prognosis for GISTs was assessed on the basis of a risk stratification scheme using the modified NIH consensus classification, which considers tumor size, mitotic count, tumor location, and tumor rupture (4).

**TABLE 1:** Clinical pathologic features of the 30 GISTs in our study cohort

Case no.	Gender	Age	Tumor location	Primary/ metastasis/ recurrence	Tumor size (cm)	Mitotic count (50 HPFs)	Risk classification	Neoadjuvant imatinib therapy	Overall survival (day)	Recurrence (recurrence-free period, days)	Prognosis (alive/ dead)	Driver gene	Amiono acid change by driver gene mutation	Group
1	M	41	Stomach	Recurrence	16	50	NA	Yes	1,470	No	alive	*KIT*	E11: p.K550_K558del	A
2	F	86	Stomach	Primary	20	20	High	No	1,336	Yes (528)	alive	*KIT*	E11: p.W557_K558del	A
3	M	74	Stomach	Primary	5	16	High	No	1,879	No	alive	*KIT*	E11: p.W557_K558delinsE	A
4	F	86	Stomach	Primary	11	20	High	No	449	Yes (4,264)	dead	*KIT*	E11: p.W557_V559delinsF	A
5	M	49	Small intestine	Primary	6	>5	High	No	506	No	alive	*KIT*	E11: p.W557_P573delinsS	A
6	F	68	Stomach	Primary	12	1	High	Yes	2,432	No	alive	*KIT*	E11: p.K550_K558del	A
7	M	78	Small intestine	Primary	9	>5	High	No	2,777	Yes (2,554)	alive	*KIT*	E11: p.E554_V560delinsV	A
8	F	66	Stomach	Primary	4	40	High	Yes	1,477	NA	dead	*KIT*	E11: p.W557_V559delinsF, E13: p.V654A	A
9	M	68	Stomach	Primary	5	28	High	No	1,825	No	alive	*KIT*	E11: p.W557_E561del	A
10	M	45	Stomach	Primary	12	2	High	Yes	2,451	Yes (5,364)	alive	*KIT*	E11: p.W557_K558del	A
11	M	86	Stomach	Primary	7	100	High	No	1,005	Yes (469)	dead	*KIT*	E11: p.P551_K558delinsQ	A
12	M	69	Stomach	Primary	6	7	High	No	753	No	alive	*KIT*	E11: p.W557_V560delinsF	A
13	M	45	Liver	Metastasis	5	3	NA	No	2,477	Yes (888)	alive	*KIT*	E11: p.Y570_L576del	B
14	F	74	Stomach	Primary	3	30	High	No	493	No	alive	*KIT*	E11: p.L576P	B
15	F	85	Stomach	Primary	4	11	High	No	1,837	No	alive	*KIT*	E11: p.V559G	B
16	F	59	Stomach	Primary	2.5	12	High	No	221	No	alive	*KIT*	E11: p.V560D	B
17	M	59	Liver	Metastasis	5	NA	NA	No	1,481	No	alive	*KIT*	E11: p.K558_T574del	B
18	M	63	Stomach	Primary	5	10	High	No	553	No	alive	*KIT*	E11: p.V560D	B
19	M	51	Small intestine	Primary	3	<5	Low	No	1,462	No	alive	*KIT*	E11: p.W557_E562del	C
20	F	64	Small intestine	Primary	3	<5	Low	No	2,882	No	alive	*KIT*	E11: p.K550_V555delinsL	C
21	M	64	Stomach	Primary	5	2	Low	No	1,796	No	alive	*KIT*	E11: p.W557_K558del	C
22	F	71	Stomach	Primary	4.5	6	Intermediate	No	39	No	alive	*KIT*	E11: p.W557_K558del	C
23	F	61	Stomach	Primary	5	3	Low	No	2,419	No	alive	*KIT*	E11: p.W557_K558del	C
24	M	65	Stomach	Primary	7	2	Intermediate	No	2,658	No	alive	*KIT*	E11: p.W557_V559delinsC	C
25	F	71	Small intestine	Primary	5	2	Low	No	1,386	No	alive	*KIT*	E11: p.W557R	D
26	F	65	Stomach	Primary	4	<5	Low	No	2,079	No	alive	*KIT*	E11: p.V559D	D
27	F	70	Stomach	Primary	5	5	Low	No	1,113	No	alive	*KIT*	E11: p.D579del	D
28	M	68	Stomach	Primary	2	2	Low	No	756	No	alive	*KIT*	E11: p.K558_E562del	D
29	M	60	Stomach	Primary	2	1	Low	No	2,017	No	alive	*KIT*	E11: p.K558_E562del	D
30	F	72	Stomach	Primary	2	1	Low	No	1,827	No	alive	*KIT*	E11: p.D579del	D

Abbreviations: M, male; F, female; HPFs, high-power fields; NA, not available.

NOTE: GIST cases were classified into the following four groups based on the presence or absence of *KIT* 557/558 deletion and the grade based on risk classification or status of metastasis/recurrence: A, high-risk or metastasis/recurrence cases with *KIT* 557/558 deletion; B, high-risk or metastasis/recurrence cases carrying a *KIT* mutation but without 557/558 deletion; C: low/intermediate-risk cases with *KIT* 557/558 deletion; D, low-risk cases carrying a *KIT* mutation but without 557/558 deletion.

### WGS

Following DNA extraction from tumor and matched peripheral blood samples, we constructed DNA libraries using a TruSeq DNA PCR-Free High Throughput Library Prep Kit (20015963; Illumina) with 1 μg DNA according to the manufacturer's instructions. Then we performed whole-genome 150-bp paired-end WGS using a NovaSeq 6000 System (Illumina). We converted the resultant raw data to FASTQ format with Bcl2fastq v2.20 (Illumina) and then used DRAGEN Bio-IT Platform v3.9 (Illumina) for mapping sequenced reads to reference human genome hs37d5, marking duplicated reads, variant calling, and calculating quality metrics.

### Variant Calling and Annotation

We classified variants as small variants, including single-nucleotide variants (SNV) and insertions or deletions Indels; (≤50 bases), or SVs (>50 bases). For SNVs and Indels, we identified somatic variants using DRAGEN Small Variant Caller. Variants with a low sequence depth in normal and/or tumor samples, as well as those with a low variant depth and/or variant allele frequency in tumor samples, were excluded from the downstream analysis. We used Ensembl Variant Effect Predictor v104 for variant annotation (21) and analyzed the drivability and actionability of variants with the in-house pipeline Shizuoka Multi-omics Analysis Protocol (SMAP; ref. 22). This pipeline evaluated and classified the annotated variants into five tiers according to the reliability of supporting information by sequentially comparing alterations among multiple databases, as described previously (22).

We detected SVs using DRAGEN Structural Variant Caller and applied the python script convertInveresion.py, which is provided as part of manta v1.6 (23), to extract inversions. Those SVs that were flagged as IMPRECISE were discarded. In addition, those for which at least one breakpoint was located within 100 bp of (i) segmental duplications, (ii) microsatellites, (iii) simple repeats, (iv) low complexity regions, and (v) ENCODE blacklist genomic regions were discarded. For each breakpoint, the gene symbol, exon/intron number, transcript accession number (RefSeq and Ensembl), and relationship to the coding exon frame were annotated by an in-house developed pipeline. SVs were categorized into five classes (translocation, insertion, deletion, duplication, and inversion) based on the mapping information for a read pair. The drivablity and actionability of SVs were assessed using SMAP. In addition to tier 1 (driver SVs) and tier 2 (likely driver SVs) annotation, SVs were annotated as tier 3 (predicted driver SVs) if they disrupted the coding sequence of a tumor suppressor gene (TSG).

### Mutational Signatures

We used the non-negative matrix factorization method to detect mutational signatures in WGS samples, and they were subsequently analyzed using MutationalPatterns v3.0.1 software (24). Briefly, a single-base substitution (SBS) profile of 96 combinations of base substitutions and neighboring bases was constructed for each sample. In the same way, an indel profile of 83 combinations of variant type, length, and sequence features was constructed for each sample. To obtain insight into the mutational signatures, these profiles were decomposed into an optimal combination of the Catalogue of Somatic Mutations in Cancer (COSMIC) signatures using the fit_to_signatures_strict function. The contribution of each signature in all samples was visualized as a heatmap.

### CNAs

CNAs were identified using the DRAGEN Copy Number Variant pipeline. Those variants that were flagged as PASS were extracted and used in the subsequent analyses. In cases where DRAGEN could not reliably estimate tumor content from the WGS dataset, those variants flagged as lowModelConfidence in the FILTER field in the variant call format (VCF) file were also extracted. Gene-specific CNs were determined by annotation against known genes in Ensembl v104. Tumors with whole-genome duplication (WGD) were defined as those where ModelSource was described as DEPTH+BAF_DOUBLED in the VCF file. The homologous recombination deficiency (HRD) score was calculated using DRAGEN HRD caller and defined as the sum of the following: (i) LOH score, (ii) telomeric allelic imbalance score, and (iii) large-scale state transition score. The CN profile of a sample was calculated as the relative length of 48 combinations of copy-number variations (CNV), and the profile was constructed from six CN categories (0, 1, 2, 3–4, 5–8, and ≥9), five CNV length categories (0–100 kb, 100 kb–1 Mb, 1–10 Mb, 10–40 Mb, and >40 Mb), and three CNV types (homozygous deletion, CNV with LOH, CNV without LOH). The profile was decomposed into COSMIC CNV signatures in the same manner as described above for mutational signatures. The ploidy score was calculated as the sum of the number of autosomal chromosome arms gained or lost.

### DNA Methylation Analysis

We generated bisulfite-converted DNA for methylation analysis using an EZ DNA Methylation Kit (D5001; Zymo Research) with 500 ng aliquots of DNA. Then, we performed a comprehensive DNA methylation analysis using the Infinium MethylationEPIC BeadChip Kit, covering 866,895 methylation sites (WG-317-1001; Illumina). Next, the specifically hybridized DNA was fluorescently labeled by a single-base extension reaction and detected using an iScan System array scanner (Illumina) in accordance with the manufacturer's protocols. Finally, the data were assembled using GenomeStudio Methylation Module v1.8 (Illumina) software. The methylation levels of the CpG sites were represented by β values ranging from 0 (completely unmethylated) to 1 (completely methylated), and the data were analyzed using the Subio Platform (https://www.subioplatform.com), Excel 2021 (Microsoft), and GraphPad Prism v8.3.0 (GraphPad Software) software. CpG sites on the sex chromosomes were excluded from the analysis, leaving 846,927 probes. To analyze the intergenic methylation levels, probes for the CpG islands around the transcription start sites (TSS) were excluded, leaving 741,629 probes. For the clustering analysis, probes whose difference was within 0.1 of the average β values in all 23 samples were excluded, leaving 648,741 probes, of which 12,000 were randomly selected using the RAND function in Excel. For global intergenic methylation analysis, 741,629 probes were binned in 300-kb lengths to determine the average β values. Methylation levels at promoter regions were assessed using the average β value of probes located on the CpG islands around TSS.

### Gene Expression Analysis

Total RNA was isolated from minced tissue samples using a miRNeasy Mini Kit (217004; Qiagen) as described previously (25). RNA samples with an RNA integrity number ≥6.0 underwent gene expression profiling (GEP) using a one-color Low Input Quick Amp Labeling Kit (5190-2305; Agilent Technologies) for amplification/fluorescence-labeling and a SurePrint G3 Human Gene Expression 8 × 60K v2 Microarray kit (G4851B; Agilent Technologies) for detecting gene expression. The microarray kit contains 50,599 probes capable of detecting 29,833 genes registered in the Entrez Gene Database of the National Center for Biotechnology Information. Hybridization signals were detected using a DNA Microarray C Scanner (Agilent Technologies), and the scanned images were analyzed using Agilent Feature Extraction v10.7.3.1 software. Microarray analysis was performed in accordance with Minimum Information About a Microarray Experiment guidelines (26). Data analysis was performed using GeneSpring GX v14.9.1 (Agilent Technologies), the Subio Platform, Excel 2021, and GraphPad Prism v8.3.0. We selected probes to be analyzed according to the reference genome sequence hg19, obtained from the UCSC Genome Browser (27). Raw signal intensity values were log_2_ transformed and normalized to the 75th percentile.

### Gene Expression Signatures

The expression signatures/scores were calculated from z-scores from the mean log_2_ expression values of the genes corresponding to each signature, including TP53 inactivation (28), chromosomal instability (CIN; ref. 29), and hypoxia (30). The genes and probes for each signature are listed in Supplementary Table S1.

### Statistical Analysis

We used Welch *t* test and Fisher exact test for comparisons between two groups and Pearson correlation coefficient to compare two variables. *P* values <0.05 were considered statistically significant.

### Data Availability

Sequencing, expression, and methylation data have been deposited in the National Bioscience Database Center (NBDC) under accession number JGAS000604. Access can be requested through the NBDC application system (https://humandbs.biosciencedbc.jp/en/data-use). Other data generated in this study are available within the article and its Supplementary Data.

## Results

### WGS

We performed WGS of 60 genomes from DNA isolated from tumor tissue and matched blood samples from 30 patients with GIST with pathogenic *KIT* mutations in exon 11 (Supplementary Fig. S1) to identify the genomic characteristics contributing to the development of *KIT* Δ557–558 in GISTs. The presence of *KIT* mutations was identified by WES, panel sequencing, Sanger sequencing, and the IGV tool, and some cases had been reported previously (16). The 30 GIST cases were classified into the following four groups according to the presence or absence of *KIT* Δ557–558 and the grade based on risk criteria using the modified NIH consensus classification (4) or status of metastasis/recurrence (Table 1; Supplementary Fig. S2): group A (12 cases), high-risk or metastasis/recurrence cases with *KIT* Δ557–558; group B (six cases), high-risk or metastasis/recurrence cases carrying a *KIT* exon 11 mutation other than *KIT* Δ557–558; group C (six cases), low/intermediate-risk cases with *KIT* Δ557–558; and group D (six cases), low-risk cases carrying a *KIT* exon 11 mutation other than *KIT* Δ557–558. No significant differences in overall survival or relapse-free survival were observed; however, group A GISTs tended to display the worst survival (Supplementary Fig. S3). WGS was performed to an average read depth of 130.3 × (range: 115.7–150.7 ×) in the tumor samples and 36.2 × (range: 28.8–45.9 ×) in the matched blood samples. The *KIT* mutations detected by WGS in all cases were identical to the mutations previously obtained by other methods. *KIT* mRNA expression was confirmed in all 30 samples by GEP data (Supplementary Table S2).

### Somatic Mutational Landscape

**FIGURE 1:**
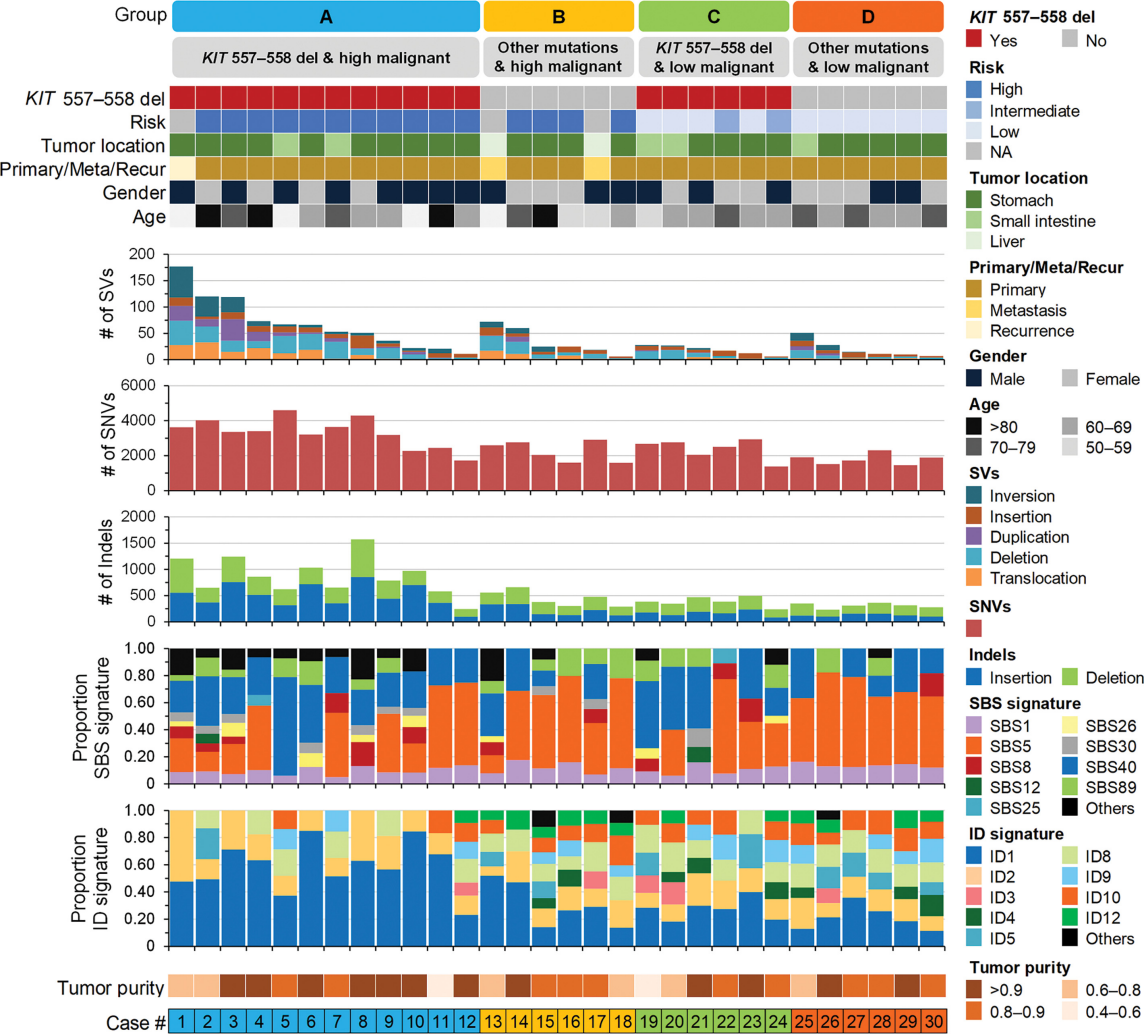
Somatic mutational landscape of the 30 GISTs in our study cohort. The top panel shows clinical information for each sample, including somatic pathogenic variant status of *KIT* Δ557–558; risk stratification using a modified Fletcher classification; tumor location; derivation of primary, metastasized, and recurring tumors, gender, and age at operation. The number of SVs, SNVs, and Indels for each sample, along with the proportion of SBS and ID signatures, are represented by bar graphs. The case number and tumor purity are shown at the bottom.

The somatic mutational landscape differed between malignant GISTs with *KIT* Δ557–558 and other cases (Fig. 1; Supplementary Figs. S1 and S4; Supplementary Table S2; Supplementary Data S1 and S2). GISTs with *KIT* Δ557–558 in group A exhibited significantly more SVs, SNVs, and Indels than other cases in group B, C, and D, respectively (*P* < 0.05), with the exception of SVs in group B. Pairs of SVs, SNVs, and Indels values were positively correlated in all 30 GISTs (0.65–0.72) and in group A GISTs (0.42–0.52).

The mutational signatures, including SBS and small insertions and deletions (ID; also known as indels), were compared with signatures from the COSMIC database (31). SBS1, SBS5, and SBS40, known as “clock-like” signatures, were predominant among all samples. SBS89, whose etiology is unknown, was detected in 57% of samples (>5% contribution). Signatures more common in malignant GISTs with *KIT* Δ557–558 (group A) included SBS8, SBS26, and SBS30 (>5% contribution). SBS8, of unknown etiology and characteristic of C > A and T > A, was detected in 12 samples (group A = 6 and group C = 3). SBS26, which is associated with defective DNA mismatch repair and characteristic of T > C, was detected in six samples, all from GISTs carrying *KIT* Δ557–558 (group A = 4 and group C = 2). SBS30, which is due to deficient base excision repair caused by inactivating mutations in *NTHL1* and characteristic of C > T, was detected in 10 samples, all from GISTs carrying *KIT* Δ557–558 (group A = 7, group B = 2, and group C = 1). These three signatures may be characteristic of *KIT* Δ557–558 in GISTs.

ID1 and ID2 contributed more than 10% in all samples except one sample with ID2. Only ID1 showed a significantly higher contribution in group A compared with the other groups (*P* < 0.0001). ID1 is characteristic of predominant insertions of thymine at thymine mononucleotide repeats. ID2 is predominantly composed of deletions of thymine at thymine mononucleotide repeats. Alexandrov and colleagues proposed that ID1 and ID2 were probably due to slippage of either the nascent (ID1) or template (ID2) strand during DNA replication at poly-T tracts (30). In addition, these signatures were found in most samples from most cancer types, but were particularly common in colorectal, stomach, endometrial, and esophageal cancers and in diffuse large B-cell lymphoma. Collectively, these molecular landscapes on the various *KIT* exon 11 mutations in GISTs indicate genomic instability in malignant GISTs with *KIT* Δ557–558.

### Somatic CNAs

**FIGURE 2:**
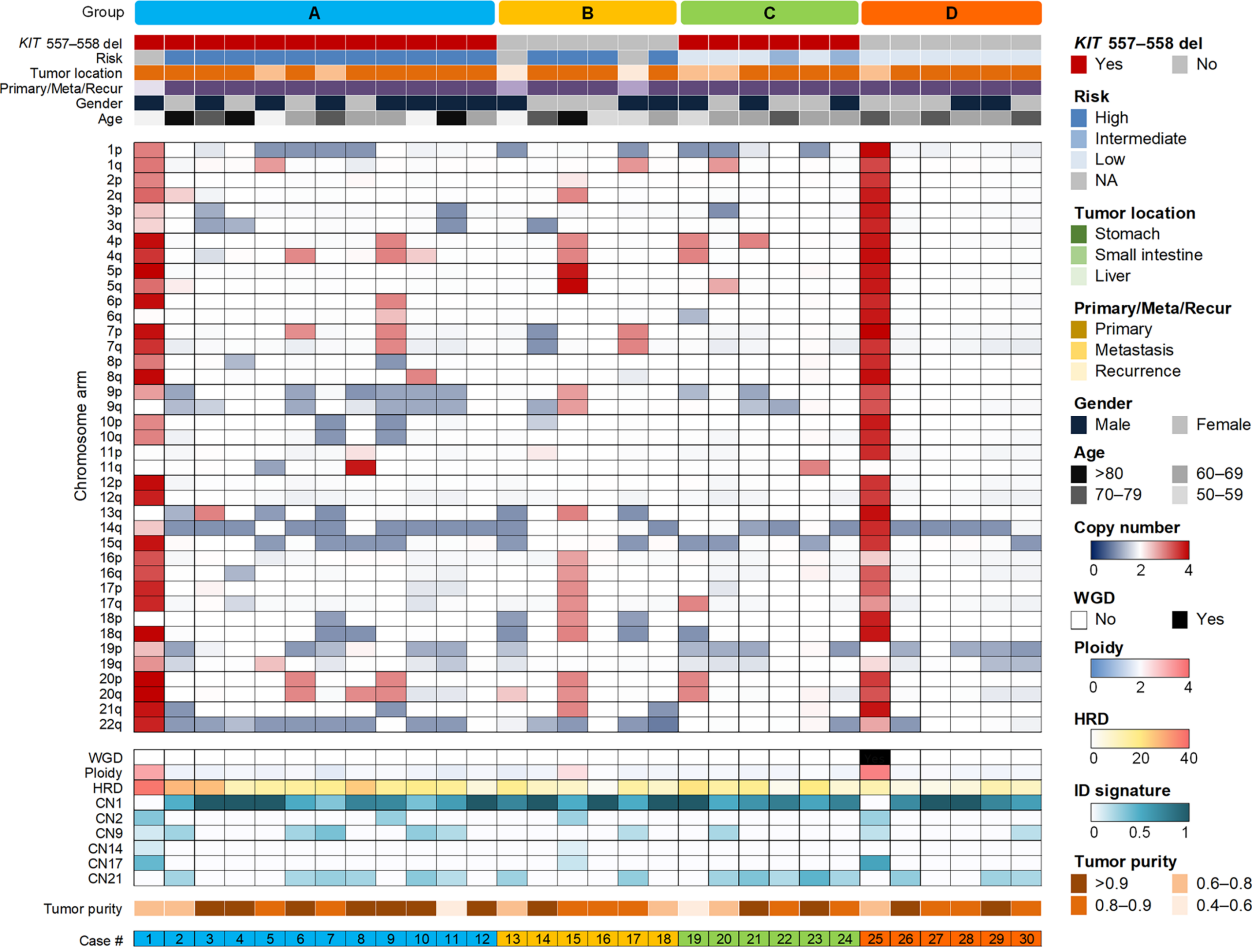
Whole-genome DNA CN profile of the 30 GISTs in our study cohort. Chromosomal arms with a gain or loss are indicated in red and blue, respectively. Only the q arm is shown for acrocentric chromosomes 13, 14, 15, 21, and 22. CN signatures are shown along with the status of WGD, ploidy, and HRD.

Next, we investigated whole-genome level CNAs in GISTs (Fig. 2; Supplementary Table S2; Supplementary Data S3). Among the 30 cases, two (nos. 1 and 25) showed CN increase across the entire region (ploidy >3), with WGD detected in case 1 and the highest HRD score in case 25. Furthermore, CN signature analysis also revealed that cases 1 and 25 showed CN17 and CN2, indicative of HRD and a tetraploid genome, respectively. CN2 was also shown in two other cases (nos. 9 and 15).

Deletions in chromosome arms 14q, 22q, 1p, 9p, and 15q have been frequently observed in GISTs, and these cytogenetic events are necessary for the malignant transformation of micro-GISTs into tumors (12). Our WGS analysis also identified these deletions (Fig. 2). The frequently deleted regions included chromosome arms 14q (60%), 22q (50%), 19p (47%), 15q (33%), 1p (30%), and 9p (27%). Excluding the two samples with higher ploidy, we compared the frequency of deletions between the malignant GISTs with *KIT* Δ557–558 (group A) and other cases (group B–D). A comparison of the average CNs revealed that chromosome arms 9p, 9q, and 22q were significantly different (*P* < 0.05, Welch *t* test). No significance (*P* < 0.05, Fisher exact test) was found in chromosome arm 9q when the number of deleted samples was compared (CN: <1.5). In addition, comparing the frequency of deletions between high-risk (group A and B) and low-risk (group C and D) GISTs revealed a significant difference (*P* < 0.05, Welch *t* test) in the average CNs in chromosome arms 3p, 19p, and 22q. Among them, only chromosome arm 22q showed a significant difference (*P* < 0.05, Fisher exact test) in the number of deleted samples. On the other hand, CN increases were observed in several samples except for the less malignant GISTs without *KIT* Δ557–558 (group D), particularly in chromosomes 4 and 20. Collectively, our findings indicated that deletions in chromosome 22q were related to the malignant progression of GISTs independent of the type of exon 11 mutation and that deletions in chromosome 9p were mainly related to malignant GISTs with *KIT* Δ557–558.

### Whole-genome Landscape of Driver Alterations

**FIGURE 3:**
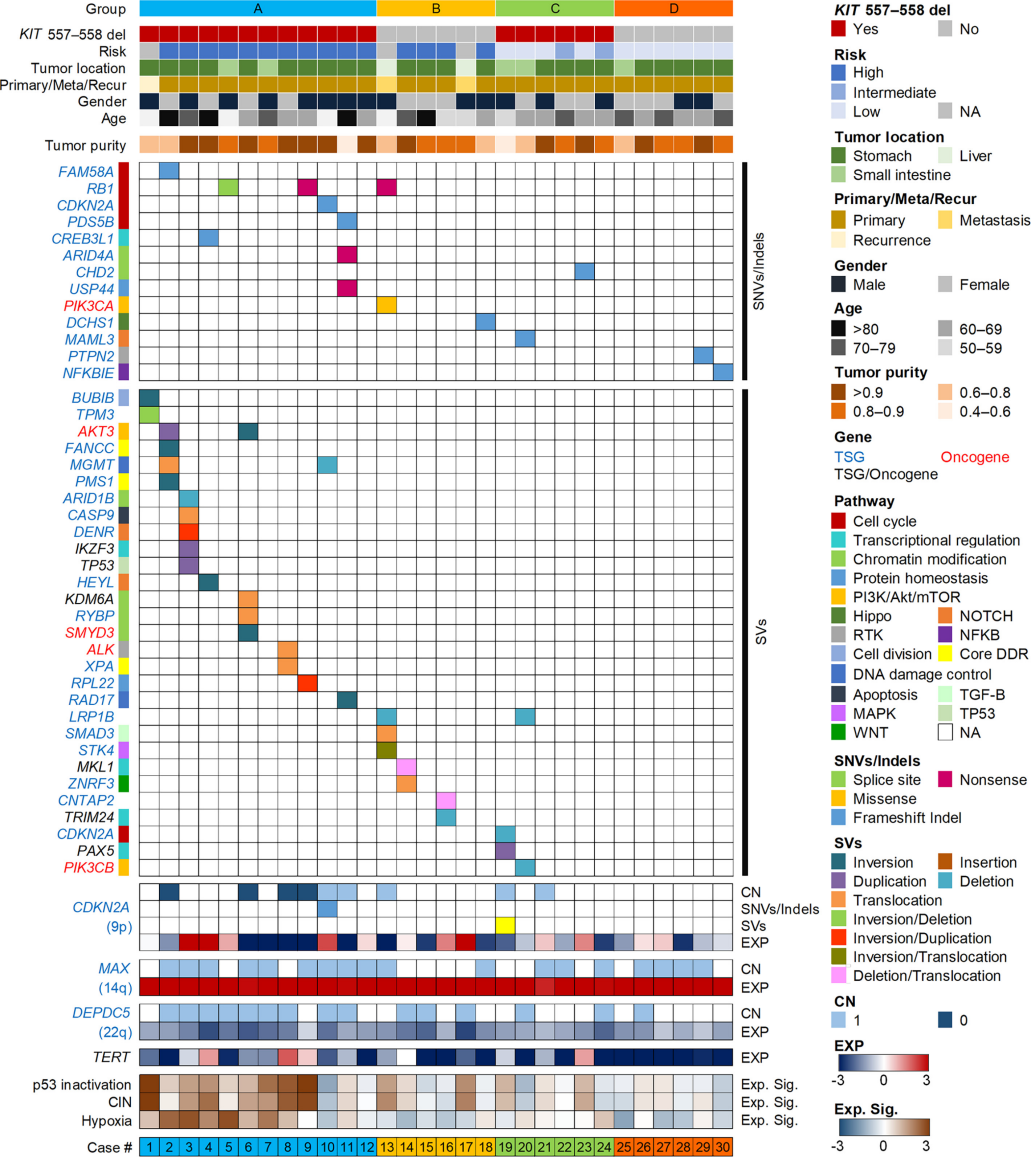
Whole-genome landscape of somatic driver alterations in the 30 GISTs in our study cohort. SNVs/Indels and SVs identified as driver alterations are shown. In *CDKN2A* (chromosome 9p), *MAX* (chromosome 14q), and *DEPDC5* (chromosome 22q), which are genes suggested as associated with CN reduction in malignant GISTs, the status of CN, SNVs/Indels, SVs, and expression (EXP) is indicated along with *TERT* expression status and expression signatures for p53 inactivation, CIN, and hypoxia.

*KIT* mutations, along with *PDGFRA* mutations, are involved in the earliest events in GIST development. As described previously in this and other studies, CNAs and other mutations have been associated with GIST progression. In particular, activation of the cell-cycle pathway by *RB1* and *TP53* mutations and the PI3K pathway by *PIK3CA* and *PTEN* mutations were suggested as involved in the malignant transformation of GISTs (15, 16). Overall, our WGS analysis revealed 15 driver gene mutations from 13 genes in 12 cases (Fig. 3). Only *RB1* mutations were recurrently detected (three cases). In the 18 malignant cases (group A and B), 11 mutations from nine genes were found in eight cases. Furthermore, eight mutations from seven genes were identified in 50% (six of 12 cases) of the malignant GISTs with *KIT* Δ557–558 and included cell cycle–related genes in five mutations from four genes (*RB1*, *CDKN2A*, *FAM58A*, and *PDS5B*). Regarding PI3K-related mutations, *PIK3CA* mutation was found in a malignant case without *KIT* Δ557–558.

Currently, there are no reports on SV detection in GISTs by comprehensive WGS analysis. In this study, we detected 1,257 SVs in all 30 cases and evaluated them as driver SVs using our in-house pipeline SMAP (Supplementary Data S1). No tier 1 pathogenic driver SVs were identified, but 42 SVs with driver potential were identified, including four tier 2 SVs and 38 tier 3 SVs. Of the 42 SVs, after excluding SVs that contained the same genes, 32 potential driver SVs from 29 genes were detected in 14 of the 30 GIST cases. The genes recurrently detected in potential driver SVs were *AKT3*, *LRP1B*, and *MGMT*. Because the number of SVs was significantly higher in the malignant cases with *KIT* Δ557–558 (Supplementary Fig. S4), potential driver SVs were detected in 75% (nine of 12 cases) of these cases (group A), in which SVs were recurrently involved in *AKT3* and *MGMT*.

As mentioned above, the deletion in chromosome 9p was characteristic of malignant GISTs with *KIT* Δ557–558 and *CDKN2A* deletion was the most notable aberration in this region. We identified a decrease in *CDKN2A* CN in six of the 12 malignant cases with *KIT* Δ557–558. Among them, loss of function was predicted in five cases due to decreased *CDKN2A* expression and in the remaining case (no. 10) due to LOH. The other five cases showed reduced *CDKN2A* mRNA expression. Decreased *CDKN2A* expression was also observed in another sample, which had no mutations or SVs in *CDKN2A*, possibly due to promoter hypermethylation (32, 33). Collectively, the loss-of-function alterations of *CDKN2A* occurred in seven of the 12 malignant cases with *KIT* Δ557–558. Two TSGs thought to be involved in GIST progression, *MAX* and *DEPDC5* on chromosome 14q and 22q, respectively (14, 34), were also investigated for alterations in CN and expression. CN decreases of *MAX* were observed in 18 of 30 cases regardless of the malignancy grade, but this did not affect its expression. CN decreases of *DEPDC5* were observed in 15 cases, including nine malignant cases with *KIT* Δ557–558, in which some showed a reduction in expression. *DMD* on chromosome Xp has been suggested as a TSG involved in GIST development (13). SVs of *DMD*, which codes for dystrophin, were observed in 12 cases, all involving deletions, 10 of which were malignant cases with *KIT* Δ557–558 (Supplementary Fig. S5). No clear relationship was observed between SVs and expression, but SVs may alter the protein structure of dystrophin, consequently influencing its function (13). Combining the results of mutations, SVs, and *CDKN2A*, 11 of 12 cases, excluding case 12, showed driver alterations.

Expression data showed that three of the four cases with elevated telomerase reverse transcriptase (*TERT*) expression were malignant cases with *KIT* Δ557–558, but a comparison of expression levels showed no significant differences (*P* = 0.053) between these cases and the others. p14^ARF^ encoded by *CDKN2A* directly interacts with MDM2 to antagonize its inhibition of p53 (35). Although TP53 mutations or SVs were not observed in the malignant cases with *KIT* Δ557–558 except in one case, the p53 inactivation signature was significantly higher (*P* = 0.019), along with the CIN signature (*P* = 0.030) and the hypoxia signature (*P* = 0.00096; Supplementary Data S4). These results indicate that genomic instability and hypoxia occurred in malignant GISTs with *KIT* Δ557–558.

### Global Intergenic Hypomethylation Specific to Malignant GISTs with *KIT* Δ557–558

**FIGURE 4:**
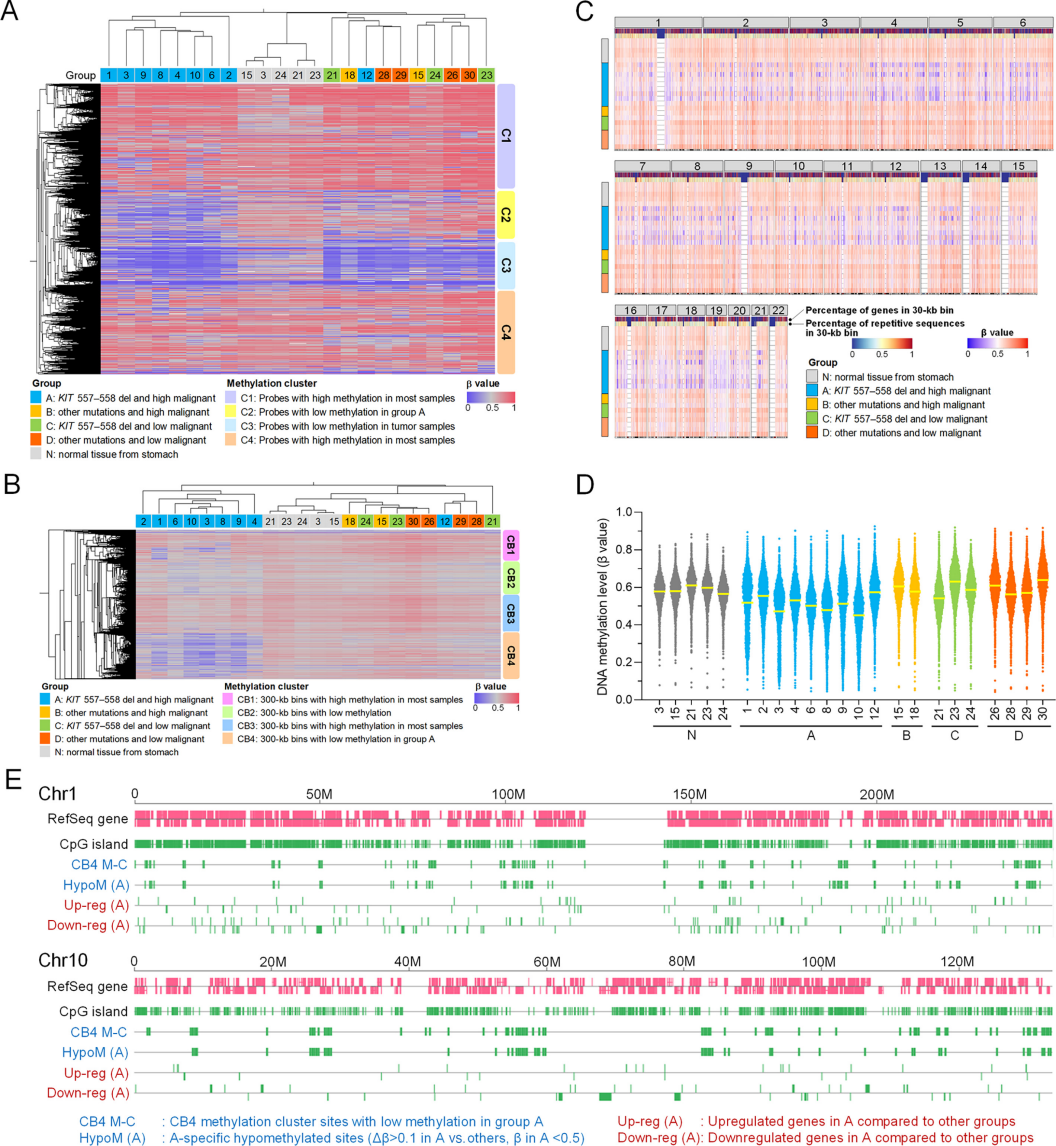
Global intergenic hypomethylation in malignant GISTs with *KIT* Δ557–558. **A,** Heatmap showing hierarchical clustering of methylation status in GISTs. Samples include 18 GISTs (group A = 9, group B = 2, group C = 3, and group D = 4) and five normal tissues (N). The RAND function in Excel was used to randomly select 12,000 probes from a total of 648,741. Prior to that, we excluded probes for sex chromosomes, probes for CpG islands around TSS, and probes whose difference was within 0.1 of the average β values in all 23 samples. Color scales indicate average β values ranging from 0 (completely unmethylated) to 1 (completely methylated). **B,** Heatmap showing hierarchical clustering of methylation status using probes binned in 300-kb lengths. **C,** Autosomal DNA methylation in GISTs. Genome-wide DNA methylation sites are shown in 300-kb bins. Low (hypomethylation) and high (hypermethylation) methylation levels are indicated in blue and red, respectively. **D,** Violin plot showing DNA methylation levels. β values in 300-kb bins were used. **E,** Genome-wide relationship between DNA methylation and gene expression in malignant GISTs with *KIT* Δ557–558. The figure shows chromosomes 1 and 10 as examples. All autosomes are shown in Supplementary Fig. S9. The first and second lines indicate the location of RefSeq genes and CpG islands, respectively. The third and fourth lines indicate the low methylation sites in group A from the CB4 methylation cluster and the group A-specific hypomethylated sites, respectively. The fifth and sixth lines indicate the sites for the group A-specific upregulated and downregulated genes, respectively.

**FIGURE 5:**
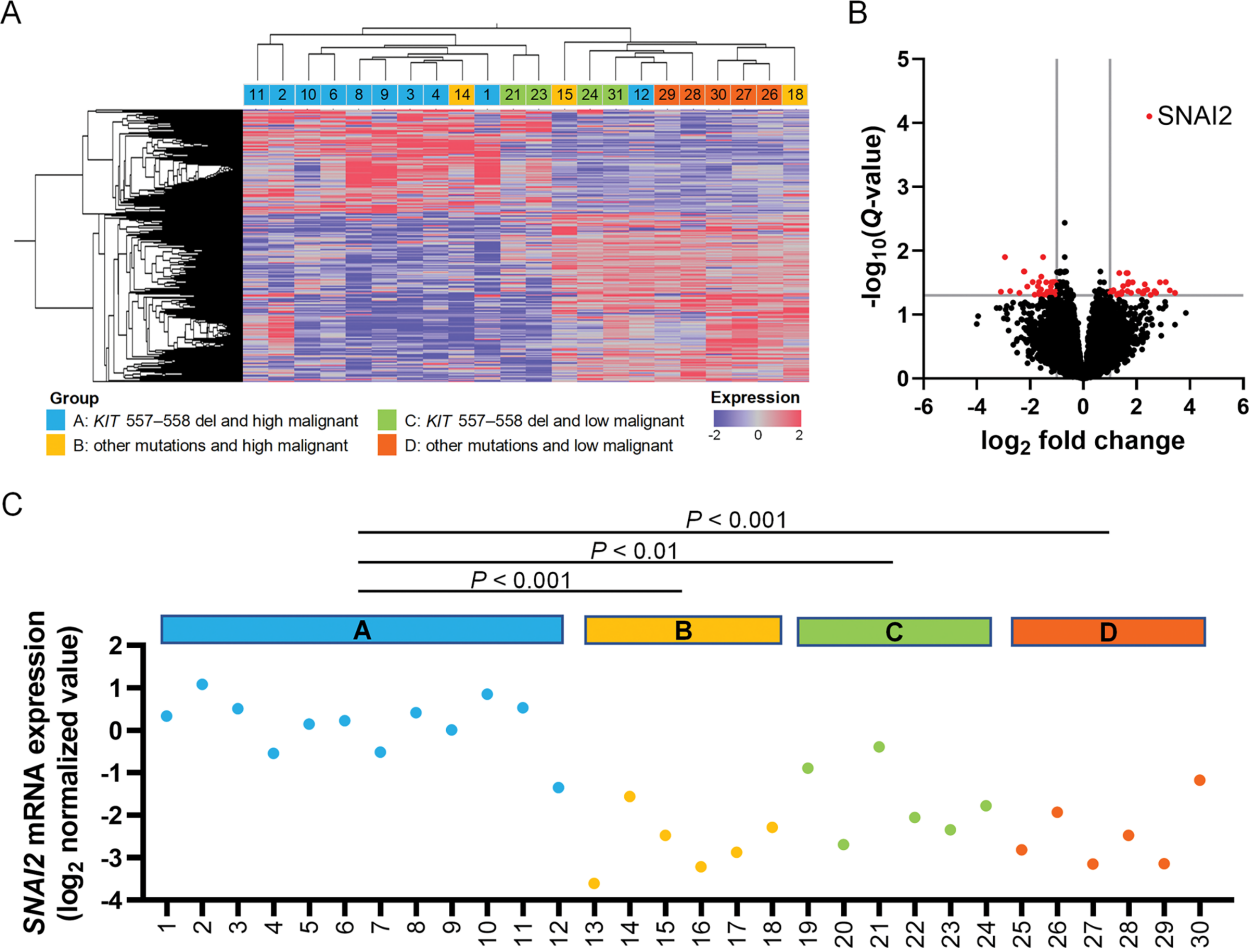
Differentially expressed genes in malignant GISTs with *KIT* Δ557–558. **A,** Heatmap showing hierarchical clustering of differentially expressed genes in malignant GISTs with *KIT* Δ557–558 (group A) compared with other cases (group B, C, and D). The case number is indicated on the top. Case 31, which is not included in the samples used for WGS or methylation analysis, has *KIT* Δ557–558 with low-risk (group C). **B,** Volcano plot showing significantly differentially expressed genes in the malignant GISTs with *KIT* Δ557–558 (group A) compared with other cases (group B, C, and D). log_2_ fold change (FC) in group A versus other groups is represented on the *x*-axis. All circles represent 25,671 microarray probes corresponding to mRNA; red circles indicate probes showing a significant expression difference (FC ≥ 1 or FC ≤ −1, *Q* < 0.05), and black circles indicate other probes. The *y*-axis shows the log_10_ of the *Q* value. A *Q* value of 0.05 and an FC of 2 are indicated by horizontal and vertical gray lines, respectively. **C,***SNAI2* mRNA expression level in the 30 GISTs in our study cohort. Each value is color coded and plotted by group. Significant differences in each group are indicated.

DNA hypermethylation at promoter regions and global hypomethylation have been observed in GISTs (17, 18). We explored methylation changes in patients with GIST malignancies by performing methylation analysis of nine group A cases, nine other cases, and five cases of normal tissue. Principal component analysis of all probes revealed a different distribution in group A compared with the other groups (Supplementary Fig. S6A). In contrast, probes for CpG islands near the TSS showed differences between tumor and normal tissues but no differences between tumors (Supplementary Fig. S6B). Clustering results for all probes showed a hypomethylation cluster (C2) specific to group A (Fig. 4A). Next, to visualize the data on chromosomes, the probes were binned in 300-kb lengths, the average β value was calculated, and the 300-kb bin data were classified by clustering (CB4 in Fig. 4B). When these results were plotted on the chromosome, hypomethylated regions were observed in group A across the entire chromosome (Fig. 4C; Supplementary Fig. S7). The 300-kb bin β values in group A tended to be lower than those in other groups (Fig. 4D). Differences in expression data also distinguished group A from other groups: 926 and 1,497 probes showed increased and decreased expression, respectively, in group A (Fig. 5A). The volcano plot displayed significant expression differences in 74 probes, 66 of which corresponded to 62 genes by annotation (Fig. 5B; Supplementary Data S5; Supplementary Table S3). Among them, *SNAI2* had the highest significance and expression differences, and a comparison of *SNAI2* expression between each group also showed significant differences (Fig. 5C). In terms of the expression (Fig. 5A) and methylation (Fig. 4A) pattern as well as *SNAI2* expression (Fig. 5C), case 12 is different from the other deletion cases and may be in a high-risk group but with a low malignant status. Gene ontology enrichment analysis identified the involvement of genes related to response to DNA damage via p53 (Supplementary Fig. S8). The genes that showed these changes, hypomethylated regions, and genes corresponding to probes that showed differences in group A were plotted on the chromosomes, clearly revealing that hypomethylation was more common in the intergenic regions (Fig. 4E; Supplementary Fig. S9).

## Discussion

Genomic instability is a hallmark of cancer (36) and is reflected by SVs, SNVs, and Indels (37), as well as DNA hypomethylation (38). Global DNA hypomethylation has been observed in malignant cases of GISTs (18), but there was no information about which exons were involved and what type of mutations were present. Among the malignant cases in this study, we found that GISTs carrying *KIT* Δ557–558 resulted in increased numbers of SVs, SNVs, and Indels, as well as global DNA hypomethylation, compared with cases carrying other exon 11 mutations (Fig. 1; Fig. 4). The p53 and CIN expression signature scores were also significantly enhanced in the malignant GISTs with *KIT* Δ557–558. This phenomenon was not observed in low-grade, less malignant GISTs with *KIT* Δ557–558. We demonstrated that *KIT* Δ557–558 mutations are associated with increased genomic instability in malignant GISTs.

Among the *KIT* exon 11 mutations, those with deletions including *KIT* Δ557–558 showed increased ERK1/2 phosphorylation, increased cyclin D expression, and inactivation by RB protein phosphorylation compared with cases with point mutations, suggesting that the deletion mutations led to increased cell proliferation (39). Increased expression of *ETV1* and *CXCR4* has been observed in cases of liver metastasis, proposing a metastatic mechanism involving CXCL12/CXCR4-mediated cell proliferation and invasion (10). Furthermore, it was proposed that activation of oncogenic cell growth factors can result in DNA damage and replication stress, leading to genomic instability in cases of sporadic cancers (40). In other words, strong oncogene activation promotes tumorigenesis, which in turn causes genetic alterations in cell cycle–related genes, including *TP53*, *CDKN2A*, and *ATM*, resulting in functional abnormalities. On the basis of these clinical and molecular characteristics, *KIT* Δ557–558 would show stronger oncogenic activity than other mutations.

To the best of our knowledge, no other driver genes have been reported to alter biological properties as observed in the current results, which were attributed to different mutation types within the same exon. Broadly speaking, Δ746–750 deletion and L858R point mutation in *EGFR*, which are common pathologic mutations in lung cancer, are found in exons 19 and 21, respectively (41). These mutations are associated with differences in sensitivity to TKIs (41, 42) and downstream signaling (43).

SVs alter gene expression (44, 45). We classified SVs as driver SVs using SMAP, an evaluation protocol constructed by us based on their association with oncogenesis (21): tier 1 includes pathogenic SVs, tier 2 includes likely pathogenic SVs, and tier 3 includes SVs that are predicted to disrupt TSG function. In this study, four tier 2 SVs and 38 tier 3 SVs were detected, three of which (*AKT3*, *LRP1B*, and *MGMT*) were recurrent. *AKT3* belongs to the AKT family of genes encoding serine/threonine protein kinases, and exhibits oncogenic transforming activity upon overexpression (46). Therefore, *AKT3* was categorized as an oncogene in our driver gene classification. Conversely, *AKT3* has been reported to inhibit cell proliferation and angiogenesis (47–50). One of the two cases of *AKT3* SVs (no. 2) involved the duplication of *AKT3* intron 2 upstream to the 57-Mb region, whereas the other case (no. 6) featured an inversion over a 2.7-Mb region that included *AKT3* intron 2 downstream to *SMYD3* intron 1. *AKT3* expression in these cases tended to be suppressed compared with that in the other cases (Supplementary Fig. S10). *LRP1B* is a member of the low-density lipoprotein receptor family and a putative TSG that is frequently inactivated to promote cell migration and invasion in various human cancers (51–55). The two cases with SVs in *LRP1B* (nos. 13 and 20) involved deletion. Notably, both deletions included the region from *LRP1B* intron 41 downstream to the *MTND1P27* pseudogene and their sizes were similar (416 and 474 kb, respectively). MGMT (*O^6^*-methylguanine-DNA methyltransferase) is a DNA repair protein that directly and specifically removes promutagenic DNA lesions, and its loss promotes carcinogenesis (55, 56). One of the two cases with SVs in *MGMT* (no. 2) involved a t(2;10)(q33;q26) translocation of *NBEAL1* (2q33.2) and *MGMT* (10q26.3), whereas the other case (no. 10) featured a 3.6-kb deletion from *MGMT* intron 3 upstream to *ADAM12* intron 3. Unlike AKT3, the SVs of these TSGs, namely *LRP1B* and *MGMT*, did not lead to significant changes in the expression of the respective genes (Supplementary Fig. S10). In this study, we performed gene expression analysis using microarray with 60-nucleotide probes that mostly recognize the 3′ ends of the transcripts. Therefore, if the probe recognizing regions are transcribed because of these deletions and translocations, gene expression could be observed. Further studies will reveal whether the potential driver SVs extracted in this study are actually involved in oncogenesis.

In addition to the strength of cell signaling, the genetic background may be involved in differences in genomic instability between malignant GISTs with *KIT* Δ557–558 and other exon 11 mutations. *KIT* exon 11 is the hotspot region in GISTs, with various types of mutations involving deletions, insertions, and substitutions. It would be interesting to investigate whether there is a genetic factor that causes and stabilizes *KIT* Δ557–558. This factor may also be involved in genomic instability by producing SVs, SNVs, Indels, and DNA hypomethylation. In the present study, the variants in the noncoding regions of the WGS data were not interpreted in detail, with the exception of the *TERT* driver mutations at the promoter region. Further detailed genomic and epigenomic analyses of the noncoding regions will reveal the regulatory elements, promoters, and enhancers that alter gene expression in *KIT* Δ557–558.

Hsp90 inhibitors significantly prolong progression-free survival in advanced GISTs (19) and were recently approved for marketing in Japan. HSP90 inhibitors exert their antitumor effects by inhibiting the stabilization of KIT and PDGFRA proteins by Hsp90 (57). Hsp90 stabilizes HIF1α and induces hypoxia (58). Hsp90 inhibitors at the C-terminus degrade HIF1α and suppress hypoxia (59). In this study, the hypoxia score of the expression signature was higher in the malignant GISTs with *KIT* Δ557–558 (Fig. 3), indicating enhanced HIF1α activity. Therefore, it will be interesting to investigate the relationship between the efficacy of Hsp90 inhibitors and hypoxia scores.

Our comparative analysis revealed the significant upregulation of *SNAI2* expression in malignant GISTs with *KIT* Δ557–558 (Fig. 5). *SNAI2* encodes the transcription factor Snai2 (formerly known as Slug), one of three members of the snail zinc finger protein family (60). Snai2 promotes tumor cell metastasis through epithelial–mesenchymal transition (EMT), and Snai2 overexpression predicts poor prognosis in patients derived from various cancer types (60). We and Ding and colleagues each previously reported the close correlation of *SNAI2* expression with high-risk or metastatic GISTs, although the type of mutation was not considered (61, 62). Cells undergoing EMT induces genomic instability via persistent proliferation (63). In addition, Snai2 is activated by HIF1α under hypoxia in the tumor microenvironment to promote metastasis (64, 65). Hypoxia occurs in many solid tumors and contributes to genomic instability via aberrant DNA damage signaling and DNA repair, resulting in increased mutation rates (66). Taken together, tumor cells undergoing EMT with elevated *SNAI2* expression and hypoxia scores may have caused genomic instability.

In this study, we performed a multi-omics analysis of WGS, genome-wide DNA methylation, and GEP data to elucidate global genomic and epigenomic abnormalities in patients with malignant GISTs carrying *KIT* Δ557–558. These results potentially provide evidence that patients with GIST with *KIT* Δ557–558 have a poor prognosis. However, this study had several limitations. First, this study had a small cohort size, and it was a single-institutional study. Second, the malignancy of GIST was evaluated using the modified NIH classification. Third, we did not completely eliminate the effect of imatinib pretreatment on the results, because of clonal selection. Finally, we did not perform functional analyses to evaluate the potential driver SVs. Therefore, further studies are needed in the future to validate these results.

## Supplementary Material

Supplementary Figure S1 Circos plots of the 30 GIST genomes in our study cohort.

Supplementary Figure S2 KIT exon 11 mutations detected in 30 GIST samples.

Supplementary Figure S3 Kaplan-Meier curves.

Supplementary Figure S4 Comparison of somatic mutational landscape between malignant and less malignant GISTs with and without KIT Δ557–558.

Supplementary Figure S5 SVs and expression levels of DMD in 30 GIST samples.

Supplementary Figure S6 Visualization of methylation status in GISTs.

Supplementary Figure S7 DNA methylation in each autosome of GISTs. Genome-wide DNA methylation sites are shown in 300-kb bins.

Supplementary Figure S8 Functional enrichment analysis of the differentially expressed genes in malignant GISTs with KIT Δ557–558.

Supplementary Figure S9 Genome-wide relationship between DNA methylation and gene expression in malignant GISTs with KIT Δ557–558.

Supplementary Figure S10 Expression levels for genes involved in recurrently detected SVs.

Supplementary Table S1 Genes for expression signatures.

Supplementary Table S2 Summary of WGS, methylation and expression data.

Supplementary Table S3 Probes for genes with specific expression alterations in malignant GISTs with KIT Δ557–558.

Supplementary Data S1 SVs identified in the 30 GIST cases in our study cohort.

Supplementary Data S2 SNVs/Indels identified in the 30 GIST cases in our study cohort.

Supplementary Data S3 CNAs identified in the 30 GIST cases in our study cohort.

Supplementary Data S4 Data for gene expression signatures.

Supplementary Data S5 Expression data of probes used for the volcano plot comparing between malignant GISTs carrying KIT Δ557–558 and other GISTs.
