# Supplementary material for: Whole-genome and Epigenomic Landscapes of Malignant Gastrointestinal Stromal Tumors Harboring KIT Exon 11 557–558 Deletion Mutations
Source: Cancer Res Commun. 2023 Apr 24;3(4):684–96. doi: 10.1158/2767-9764.CRC-22-0364 (PMC10124575; doi:10.1158/2767-9764.CRC-22-0364)
Supplement: Supplementary Figure S1 — Circos plots of the 30 GIST genomes in our study cohort. [file crc-22-0364-s01.docx]

**Supplementary Fig. S1.** Circos plots of the 30 GIST genomes in our study cohort. **A,** Circos plots of 12 GIST genomes harboring *KIT* Δ557–558 with high-risk or recurrence. **B,** Circos plots of 6 GIST genomes harboring other *KIT* exon 11 mutations with high-risk or recurrence. **C,** Circos plots of 6 GIST genomes harboring *KIT* Δ557–558 with low- or intermediate-risk. **D,** Circos plots of 6 GIST genomes harboring other *KIT* exon 11 mutations with low-risk. Numbers shown on each plot are the case number. The outermost (first) ring represents ideograms of human chromosomes (chromosomes 1 to y; centromeres, red line), with the pter-qter orientation in a clockwise direction. The second ring shows variant allele frequencies of single nucleotide variants. The third ring represents somatic copy-number gains (yellow-red gradient) and losses (blue gradient). The innermost area represents breakpoints of acquired genomic rearrangements (≥1 kb), with their links colored according to the following variant types: deletions (blue), tandem duplications (red), inversions (gray), and interchromosomal translocations (green). Chromosome rearrangements in which both break points are located within the genes are indicated by thick lines.
