# Supplementary material for: Whole-genome and Epigenomic Landscapes of Malignant Gastrointestinal Stromal Tumors Harboring KIT Exon 11 557–558 Deletion Mutations
Source: Cancer Res Commun. 2023 Apr 24;3(4):684–96. doi: 10.1158/2767-9764.CRC-22-0364 (PMC10124575; doi:10.1158/2767-9764.CRC-22-0364)
Supplement: Supplementary Figure S2 — KIT exon 11 mutations detected in 30 GIST samples. [file crc-22-0364-s04.docx]

**Supplementary Fig. S2.** *KIT* exon 11 mutations detected in the 30 GIST cases in our study cohort. **A,** Codon location of *KIT* exon 11 mutants. Amino acid changes were shown in orange for deletions, yellow for deletions with an insertion, and blue for a substitution. **B,** Nucleotide sequence of *KIT* exon 11 mutants. Nucleotide changes were shown in orange for deletions, in yellow for deletions with a missense mutation, and in blue for a missense mutation.
