# Supplementary material for: Whole-genome and Epigenomic Landscapes of Malignant Gastrointestinal Stromal Tumors Harboring KIT Exon 11 557–558 Deletion Mutations
Source: Cancer Res Commun. 2023 Apr 24;3(4):684–96. doi: 10.1158/2767-9764.CRC-22-0364 (PMC10124575; doi:10.1158/2767-9764.CRC-22-0364)
Supplement: Supplementary Figure S3 — Kaplan-Meier curves. [file crc-22-0364-s05.docx]

**Supplementary Fig. S3.** Kaplan-Meier curves. Overall survival curves (OS, left) and relapse-free survival curves (RFS, right) for the subgroups (A–D). The log-rank test (Mantel-Cox) was used to determine the significance of OS and RFS among the subgroups.
