# Supplementary material for: Whole-genome and Epigenomic Landscapes of Malignant Gastrointestinal Stromal Tumors Harboring KIT Exon 11 557–558 Deletion Mutations
Source: Cancer Res Commun. 2023 Apr 24;3(4):684–96. doi: 10.1158/2767-9764.CRC-22-0364 (PMC10124575; doi:10.1158/2767-9764.CRC-22-0364)
Supplement: Supplementary Figure S4 — Comparison of somatic mutational landscape between malignant and less malignant GISTs with and without KIT Δ557–558. [file crc-22-0364-s06.docx]

**Supplementary Fig. S4.** Comparison of somatic mutational landscape between malignant and less malignant GISTs with and without *KIT* Δ557–558. **A**, Comparison of number of SVs. **B**, Comparison of number of SNVs. **C**, Comparison of number of indels. Significance was indicated only for pairs with significant differences (*P*<0.05).
