# Supplementary material for: Whole-genome and Epigenomic Landscapes of Malignant Gastrointestinal Stromal Tumors Harboring KIT Exon 11 557–558 Deletion Mutations
Source: Cancer Res Commun. 2023 Apr 24;3(4):684–96. doi: 10.1158/2767-9764.CRC-22-0364 (PMC10124575; doi:10.1158/2767-9764.CRC-22-0364)
Supplement: Supplementary Figure S5 — SVs and expression levels of DMD in 30 GIST samples. [file crc-22-0364-s07.docx]

**Supplementary Fig. S5.** SVs and expression levels of DMD in the 30 GIST cases in our study cohort.
