# Supplementary material for: Whole-genome and Epigenomic Landscapes of Malignant Gastrointestinal Stromal Tumors Harboring KIT Exon 11 557–558 Deletion Mutations
Source: Cancer Res Commun. 2023 Apr 24;3(4):684–96. doi: 10.1158/2767-9764.CRC-22-0364 (PMC10124575; doi:10.1158/2767-9764.CRC-22-0364)
Supplement: Supplementary Figure S6 — Visualization of methylation status in GISTs. [file crc-22-0364-s08.docx]

**Supplementary Fig. S6.** Visualization of methylation status in GISTs. **A,** PCA using all probes. **B,** PCA using probes located on CpG islands around TSS. Samples include 18 GISTs (group A = 9, group B = 2, group C = 3, and group D = 4), and five normal tissues (N).
