# Supplementary material for: Whole-genome and Epigenomic Landscapes of Malignant Gastrointestinal Stromal Tumors Harboring KIT Exon 11 557–558 Deletion Mutations
Source: Cancer Res Commun. 2023 Apr 24;3(4):684–96. doi: 10.1158/2767-9764.CRC-22-0364 (PMC10124575; doi:10.1158/2767-9764.CRC-22-0364)
Supplement: Supplementary Figure S7 — DNA methylation in each autosome of GISTs. Genome-wide DNA methylation sites are shown in 300-kb bins. [file crc-22-0364-s09.docx]

**Supplementary Fig. S7.** DNA methylation in each autosome of GISTs. Genome-wide DNA methylation sites are shown in 300-kb bins. Low (hypomethylation) and high (hypermethylation) methylation are indicated in blue and red, respectively. Samples include 18 GISTs (group A = 9, group B = 2, group C = 3, and group D = 4), and five normal tissues (N).
