# Supplementary material for: Whole-genome and Epigenomic Landscapes of Malignant Gastrointestinal Stromal Tumors Harboring KIT Exon 11 557–558 Deletion Mutations
Source: Cancer Res Commun. 2023 Apr 24;3(4):684–96. doi: 10.1158/2767-9764.CRC-22-0364 (PMC10124575; doi:10.1158/2767-9764.CRC-22-0364)
Supplement: Supplementary Figure S8 — Functional enrichment analysis of the differentially expressed genes in malignant GISTs with KIT Δ557–558. [file crc-22-0364-s10.docx]

**Supplementary Fig. S8.** Functional enrichment analysis of the differentially expressed genes in malignant GISTs with *KIT* Δ557–558. Among the 74 probes showing significance for expression difference, 62 genes for 66 probes were annotated and applied to Metascape pathway and process enrichment analysis by Metascape.
