# Supplementary material for: Whole-genome and Epigenomic Landscapes of Malignant Gastrointestinal Stromal Tumors Harboring KIT Exon 11 557–558 Deletion Mutations
Source: Cancer Res Commun. 2023 Apr 24;3(4):684–96. doi: 10.1158/2767-9764.CRC-22-0364 (PMC10124575; doi:10.1158/2767-9764.CRC-22-0364)
Supplement: Supplementary Figure S9 — Genome-wide relationship between DNA methylation and gene expression in malignant GISTs with KIT Δ557–558. [file crc-22-0364-s11.docx]

**Supplementary Fig. S9.** Genome-wide relationship between DNA methylation and gene expression in malignant GISTs with *KIT* Δ557–558. The first and second lines indicate the location of RefSeq genes and CpG islands, respectively. The third and fourth lines indicate the low methylation sites in group A from the CB4 methylation cluster (Fig. 4B) and the group A-specific hypomethylated sites, respectively. The fifth and sixth lines indicate the sites for the group A-specific upregulated and downregulated genes, respectively.
