# Supplementary material for: Whole-genome and Epigenomic Landscapes of Malignant Gastrointestinal Stromal Tumors Harboring KIT Exon 11 557–558 Deletion Mutations
Source: Cancer Res Commun. 2023 Apr 24;3(4):684–96. doi: 10.1158/2767-9764.CRC-22-0364 (PMC10124575; doi:10.1158/2767-9764.CRC-22-0364)
Supplement: Supplementary Figure S10 — Expression levels for genes involved in recurrently detected SVs. [file crc-22-0364-s12.docx]

**Supplementary Fig. S10.** Expression levels for genes involved in recurrently detected SVs.
